# Allopurinol and Alkaline Phosphatase Levels in Patients with Non-Dialysis CKD

**DOI:** 10.3390/jcm15124685

**Published:** 2026-06-17

**Authors:** Hulya Taskapan, Tolgay Taskapan, Antonio Bellasi, Paul Tam, Tabo Sikaneta

**Affiliations:** 1 Nephrology Department, University of Toronto, Toronto, ON M5G 2C4, Canada; 2 Research Department, Kidney Life Sciences Institute, Toronto, ON M5G 2N2, Canada; 3 Nephrology Department, Ente Ospedaliero Cantonale, 6900 Lugano, Switzerland; 4 Nephrology Department, Scarborough Health Network, Toronto, ON M1P 2V5, Canada; 5 Department of Family and Community Medicine, University of Toronto, Toronto, ON M5S 1A1, Canada

**Keywords:** alkaline phosphatase, allopurinol, bone mineral metabolism, chronic kidney disease, fibroblast growth factor-23, parathyroid hormone, urate, vascular calcification, xanthine oxidase inhibition

## Abstract

**Background**: Inhibition of xanthine oxidase with allopurinol increases bone alkaline phosphatase (ALP) in vitro, while ALP promotes bone and vascular mineralization and is linked to reduced survival in general and renal populations. However, clinical associations between allopurinol use and ALP have not yet been reported. We examined prospective associations between allopurinol use, ALP levels, and death in patients with non-dialysis CKD. **Population and Setting**: A total of 1636 multi-ethnic patients enrolled in the CAN AIM to PREVENT, a 3-year prospective observational study in Toronto, Canada. **Primary Outcomes**: ALP levels and the combination of renal and patient deaths. **Statistical Analyses**: Joint generalized structural equation modeling, causal mediation, and controlled direct effects. **Results**: Allopurinol use was associated with increased ALP (18.3% (95% CI: 10.9, 26.2, *p* < 0.001)) but not renal and patient death (HR = 1.32 (0.87, 1.99, *p* = 0.193)). The association between allopurinol and ALP was most pronounced when PTH and urate were low, and was partially mediated by PTH (7.9%, (*p* = 0.006)). **Limitations**: Observational and post hoc designs prevent causal inferences. Lack of data about specific ALP isoforms. **Conclusions**: Allopurinol use was associated with increased ALP but not renal or patient deaths in patients with non-dialysis CKD. Future studies can confirm the generalizability to other clinical populations and examine mechanisms and clinical significance.

## 1. Introduction

The bone-specific isoform of alkaline phosphatase (BALP) facilitates bone and blood vessel mineralization by hydrolyzing pyrophosphate, a major inhibitor of mineralization [1,2]. It is primarily expressed in osteoblasts, chondrocytes, and calcifying vascular smooth muscle cells [1,2]. In healthy individuals, bone and liver isoforms of ALP coexist in serum in a 1:1 ratio, and collectively account for over 90% of total ALP activity [3]. In the absence of liver disease, changes in total ALP levels are often hypothesized to reflect BALP activity, making total ALP a potentially useful, albeit indirect, substitute biomarker of bone turnover [1,4]. Elevated ALP levels in the general, and especially renal populations, are associated with increased vascular calcification, cardiovascular complications, and mortality [5,6,7,8].

Current guidelines concerned with bone and mineral metabolism in patients with chronic kidney disease (CKD) recommend assessing bone turnover with serum parathyroid hormone (PTH) and ALP measurements, as these collectively enhance the sensitivity and specificity of histopathological diagnoses [9,10,11]. However oxidative stress can also influence bone metabolism. Xanthine oxidase (XO) generates reactive oxygen species (ROS) that inhibit osteoblast differentiation, reduce osteogenic marker expression, and promote osteoclast-mediated bone resorption [12,13,14,15]. Additionally, inflammatory cytokines such as TNFα and IL-1β upregulate XO activity in osteoblasts [13] exacerbating bone loss.

Allopurinol is a well-established XO inhibitor commonly used to treat gout in patients with CKD. Pre-clinical studies have shown reduced bone resorption, enhanced osteoblast differentiation, increased ALP, and new bone formation following treatment with allopurinol [15,16]. However, to our knowledge, relationships between allopurinol use and ALP have not been examined in any clinical setting. Here we report on prospective associations between allopurinol use, ALP and renal or patient death in a large multi-ethnic cohort of patients with non-dialysis CKD. We then report our results of exploratory analyses of changes in other mineral bone metabolism markers in response to allopurinol use.

## 2. Materials and Methods

### 2.1. Study Design and Participants

Post hoc analysis was conducted on the subset of CAN AIM to PREVENT participants with eGFRs of 15–60 mL/min/1.73 m^2^, who were not prescribed febuxostat or lipid-lowering agents (which can also reduce ALP), and who did not have evidence of liver disease at study enrollment [17].

The CAN AIM to PREVENT was an investigator-initiated, prospective, open observational cohort study conducted at three predialysis clinics in Toronto, Canada, between 2010 and 2015. The primary objective of the original study was to assess the association between inflammatory markers and progression to dialysis. Eligible participants included individuals aged 18 or older with a 2012 CKD-EPI estimated glomerular filtration rate (eGFR) below 60 mL/min/1.73 m^2^, without prior history of dialysis or renal transplantation, the absence of terminal illness, and the ability to provide informed consent. Participants were recruited by their nephrologists, and baseline demographic, clinical, and laboratory data were collected. Follow-up data, including medication use, vital signs, and laboratory values (e.g., urate and bone markers), were systematically recorded every six months for up to three years. The study was registered on ClinicalTrials.gov (NCT01974713) and approved by the institutional ethics review board. All participants provided written informed consent at enrollment, and no additional consent was required for the current analysis. All methods were performed in accordance with the relevant guidelines and regulations.

### 2.2. Exposures

Allopurinol use (yes or no), recorded at each six-monthly visit.

### 2.3. Outcome Measurements

The primary outcome was the combination of changes in ALP levels, renal and patient death. Exploratory analyses examined relationships between allopurinol, PTH, FGF23 and ALP.

### 2.4. Statistical Analysis

All statistical analyses were performed using Stata 18 (StataCorp LP, College Station, TX, USA). Baseline characteristics were summarized using descriptive statistics, with means and standard deviations, or medians and interquartile ranges, or frequencies and percentages as appropriate. Differences were assessed using *t*-tests or Wilcoxon rank-sum tests for continuous variables and chi-square tests for categorical variables, with statistical significance defined as a two-sided *p*-value < 0.05. Non-normally distributed variables, including ALP, PTH, FGF23, 25-hydroxyvitamin D (25(OH)D), body mass index (BMI), and C-reactive protein (CRP), were naturally log-transformed to approximate normality for modeling.

To account for the complexities of longitudinal data and the competing risk of informative dropout (due to death or dialysis), we employed joint generalized structural equation models (GSEMs). This approach allowed us to simultaneously model changes in repeated biomarker measurements (e.g., ALP) and time-to-event clinical outcomes (renal and patient death). Robust standard errors were derived to account for within-subject correlations inherent in longitudinal repeated measures data.

General covariates considered in assessing continuous outcomes were aspartate aminotransferase (AST), FGF23, 25(OH)D, calcium, phosphate, eGFR, CRP, age, sex, baseline diabetes status, BMI, vitamin D supplementation status, season (winter, spring, summer, autumn), patient-identified ethnicity (White, Black, South Asian, Southeast Asian, and East Asian), and visit number (used as a proxy for time, including a quadratic term of visit number to capture non-linear trends over follow-up). General covariates for the time-to-event sub-model included both time-varying and baseline variables. Time-varying covariates, recorded at each six-monthly visit and thereby capturing changes throughout follow-up, comprised ALP, PTH, FGF23, phosphate, eGFR, urate, CRP, BMI, and allopurinol use. Fixed baseline covariates included age, sex, ethnicity, and the presence of congestive heart failure, peripheral arterial disease, cerebrovascular disease, coronary artery disease, hypertension, or diabetes mellitus at the study enrollment.

Four distinct GSEMs were created—two for examining the primary outcome, and two for use in exploratory analyses:

**Model 1** focused on the natural log of ALP as the continuous independent outcome, with key predictors including urate, allopurinol use, and the interaction terms between allopurinol use and continuous natural log PTH, as well as between allopurinol use and urate. This model incorporated all general covariates for continuous outcomes. The corresponding time-to-event sub-model for patient and renal death included natural log ALP (predicted value), allopurinol use, urate, and natural log PTH as key predictors, alongside all general covariates for time-to-event outcomes.

**Model 2** mirrored Model 1 but categorized PTH into tertiles, using these tertiles and their interaction with allopurinol use as predictors for natural log ALP in the continuous outcome sub-model, while retaining the same structure regarding general covariates and the time-to-event sub-model. The parallel use of continuous PTH in Model 1 and tertile-categorized PTH in Model 2 served as a mutual sensitivity check for the allopurinol–PTH interaction across different PTH specifications.

**Model 3** investigated natural log PTH as the continuous dependent outcome, with urate, allopurinol use, and their interaction serving as key predictors. This specific continuous outcome sub-model also included natural log ALP, in addition to the general covariates for continuous outcome. Its time-to-event sub-model was structured identically to Model 1, using natural log ALP, allopurinol use, urate, and natural log PTH as key predictors along with all general covariates for time-to-event outcomes.

**Model 4** examined natural log FGF23 as the continuous dependent outcome, with predictors being urate, allopurinol use, their interaction, and natural log PTH, alongside the general covariates for continuous outcomes except allopurinol use and log PTH interaction. The time-to-event component was consistent with Model 1’s structure.

To assess robustness to potential hepatic confounding of total serum ALP, a sensitivity analysis was conducted for Models 1 and 2 by restricting the analytic sample to observations with AST within the conventional upper limit of normal (≤40 U/L), excluding 361 observations (3.6%) and yielding a sample of 9799 observations with 118 composite events. Both models were re-estimated using identical covariate structures and estimation options as the primary analyses (Supplementary Table S1).

Differences in bone markers were expressed as percentage changes [(e^β^ − 1) × 100], and time-to-event outcomes as hazard ratios (HR; e^β^). An antilog transformation was performed to obtain the actual PTH values using the formula: PTH = e^ln(PTH)^ when required.

Although a propensity score-matching approach was initially considered to address potential confounding by indication for allopurinol use, it was ultimately not pursued. This decision was due to an insufficient region of common support (a severe lack of overlap) in the propensity score distributions between allopurinol users and non-users, along with persistent significant imbalances in critical baseline covariates (such as serum urate levels and sex).

### 2.5. Mediation Analyses

To determine allopurinol’s direct and indirect effects on ALP, mediation analysis was performed. The Baron and Kenny approach, as provided in the structural equation modeling package by Stata 18, was used. This approach assumes no unmeasured confounding of the mediator–outcome relationship; results were therefore interpreted as indicative rather than definitive. This was repeated for exploratory analyses of allopurinol’s effects on urate, PTH, and 25(OH)D. Mediation proportions were reported as percentages.

To robustly explore potential mechanistic pathways, exploratory causal mediation analysis was then used to estimate natural indirect, natural direct, total effects, and proportion mediated effects of allopurinol on ALP and PTH. Robust standard errors were employed, and controlled direct effects were computed for predefined mediator levels. Effects were presented as percentages. A schematic flowchart to clarify the research design and data analysis workflow was given in Supplementary Materials (Figure S1).

## 3. Results

### 3.1. Participant Selection

A total of 2254 patients enrolled in the CAN AIM to PREVENT. After excluding 284 participants receiving febuxostat or lipid-lowering agents and 331 with a history of liver disease, 1636 participants (10,160 observations) were included. Baseline characteristics are presented in Table 1. At baseline, 388 participants (23.7%) were prescribed allopurinol; an additional 35 initiated allopurinol during follow-up.

### 3.2. Associations Between Allopurinol and ALP

**Figure 1:**
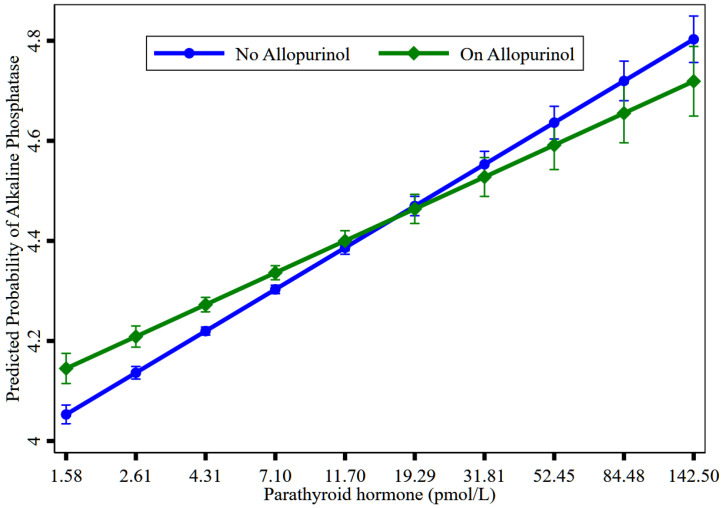
Predicted probabilities of alkaline phosphatase: Based on Generalized Structural Equation Model predicting changes in serum ALP adjusted for seasonality, Aspartate Aminotransferase, C-reactive protein, body mass index, sex, ethnicity, eGFR, age, diabetes status, visit number (used as a proxy for time), vitamin D usage, and the quadratic term of visit number. An antilog transformation was performed to obtain the actual parathyroid hormone values using the formula: PTH = e^ln(PTH)^. PTH: Parathyroid Hormone.

**Model 1:** Allopurinol use was associated with an 18.3% increase in ALP (*p* < 0.001), independent of urate and bone mineral metabolism markers. PTH and FGF23 were also positively associated with ALP (each *p* < 0.001). Negative interactions were observed between allopurinol and PTH (*p* = 0.001) and between allopurinol and urate (*p* = 0.044). Serum 25(OH)D showed no association with ALP (*p* = 0.114). Full estimates and confidence intervals are presented in Table 2; Figure 1 illustrates the relationship across PTH levels.

**Figure 2:**
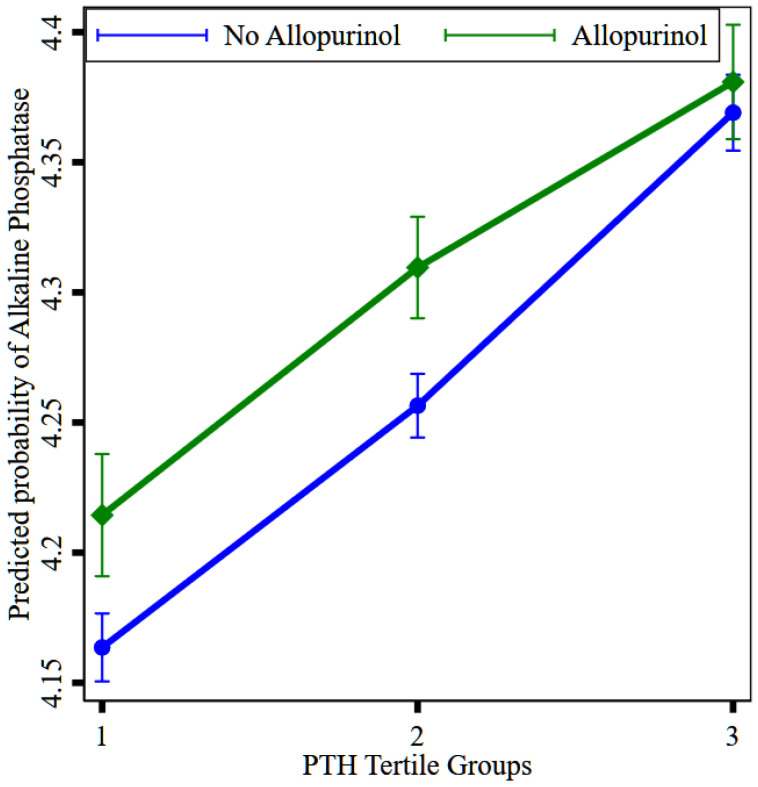
Predicted probabilities of alkaline phosphatase according to Parathyroid hormone tertile groups: Based on Generalized Structural Equation Model predicting changes in serum ALP adjusted for seasonality, Aspartate Aminotransferase, C-reactive protein, body mass index, sex, ethnicity, eGFR, age, diabetes status, visit number (used as a proxy for time), vitamin D usage, and the quadratic term of visit number. PTH: Parathyroid Hormone.

**Model 2:** PTH tertile cutoffs were determined based on the sample distribution of PTH (first tertile: <4.2 (pmol/L), second tertile: 4.2–6.8 (pmol/L), third tertile: >6.8 (pmol/L)). Higher PTH tertiles (2 and 3) were associated with progressively elevated ALP (both *p* < 0.001), with a significant negative interaction between allopurinol and the highest PTH tertile (*p* = 0.031). The interaction between allopurinol and urate remained significant (*p* = 0.037). FGF23 continued to be associated with higher ALP (*p* < 0.001). Full estimates are provided in Table 2; Figure 2 displays the tertile-stratified pattern.

### 3.3. Associations Between Allopurinol and the Combination of Renal and Patient Death

**Model 1:** Allopurinol was not significantly associated with renal and patient death (HR = 1.32, *p* = 0.193). Both ALP (HR of 2.09, *p* = 0.007) and FGF23 (HR of 2.24, *p* < 0.001)) were associated with increased mortality risk. Urate (HR 1.00, *p* = 0.988), PTH (HR 1.07, *p* = 0.690) and 25(OH)D (HR 0.93, *p* = 0.748) were not significantly associated with mortality (Table 2).

**Model 2:** Allopurinol remained unassociated with mortality (HR 1.29, *p* = 0.238), while ALP (HR 2.01, *p* = 0.012) and FGF23 (HR 2.23, *p* < 0.001) retained their associations. No gradient was observed across PTH tertiles (Table 2).

Sensitivity analyses restricted to participants with AST ≤ 40 U/L (*n* = 9799) yielded results consistent with the primary analyses across both models, with the associations between allopurinol use and ALP, the allopurinol–PTH interaction, and the relationship between ALP and the composite endpoint remaining statistically significant and of comparable magnitude (Supplementary Table S1).

### 3.4. Exploratory Analyses

**Model 3:** Allopurinol was associated with an increase in PTH levels by 27.05% (*p* < 0.001). Urate was positively associated with PTH, with a 0.06% increase per unit increase in urate (*p* < 0.001). The interaction between allopurinol and urate showed a significant inverse relationship with PTH, with a −0.05% change (*p* < 0.001). FGF23 was associated with increased PTH (13.69%, *p* < 0.001), whereas 25(OH)D was inversely associated, showing a −29.03% change (*p* < 0.001) (Table 3).

**Model 4:** Allopurinol did not significantly associate with FGF23 levels (3.32%, *p* = 0.552). Urate was positively associated with FGF23, with a 0.03% increase per unit increase in urate (*p* = 0.001). The interaction between allopurinol and urate was not statistically significant for FGF23 (−0.01%, *p* = 0.413). PTH was positively associated with FGF23 (20.99%, *p* < 0.001), while 25(OH)D was positively associated with a 5.63% increase (*p* = 0.001) in FGF23 (Table 3).

### 3.5. Exploratory Mediation and Controlled Direct Effect Analyses

Urate showed a small, non-significant indirect effect on ALP (*p* = 0.077), with a mediation proportion of 12% (*p* = 0.085). PTH demonstrated a significant indirect effect (*p* = 0.007), accounting for 7.9% of mediation (*p* = 0.006). Urate had a significant inverse indirect effect on PTH (*p* = 0.001); as the indirect and direct effects were in opposing directions, the proportion mediated was not calculated (Table 4).

To explore how the association of allopurinol with ALP varies across different levels of PTH and urate, we selected cutoffs based on the distribution of these biomarkers in our cohort such as the 10th, 25th, 50th, 75th, and 90th percentiles. Allopurinol’s association with ALP was strongest at lower PTH levels (2.69 pmol/L, corresponding to the 10th percentile: 8.5%, *p* < 0.001) and progressively declined at higher levels (11.9 pmol/L, 90th percentile: 2.8%, *p* = 0.022). Similarly, for urate, allopurinol’s association with ALP was most pronounced at lower urate levels (298 μmol/L, 10th percentile: 10.90%, *p* < 0.001) and weakened at higher levels (507 μmol/L, 75th percentile: 3.74%, *p* = 0.001). When examining PTH as an outcome, the association of allopurinol was significant at lower urate levels (298 μmol/L: 24.1%, *p* < 0.001) but was not statistically significant at higher urate levels (507 μmol/L: 1.1%, *p* = 0.579) (Table 5).

## 4. Discussion

To our knowledge, this is the first clinical demonstration of an association with ALP levels and treatment with allopurinol. Allopurinol’s association with ALP was more pronounced at lower urate levels, a pattern consistent with, though not confirmatory of, an XO inhibition-related mechanism. The positive association between allopurinol and ALP was more pronounced at lower PTH concentrations and reduced at higher PTH concentrations.

Our clinical findings are compatible with pre-clinical data on XO inhibition and bone metabolism, though the absence of bone-specific measurements precludes mechanistic conclusions. XO inhibition reduces reactive oxygen species [12,13,14], thus preventing their impairment of osteoblast function and their promotion of osteoclast activity [12,13]. Studies by Orriss et al. [16] and Laçin et al. [15] have demonstrated that allopurinol treatment enhances osteoblast differentiation and activity, promotes new bone formation, and reduces bone resorption in experimental models. This was complemented by a substantial rise in tissue non-specific alkaline phosphatase (TNAP) activity—up to 65% in vitro—as well as increased TNAP mRNA and osteocalcin expression [16], potentially by shifting the inorganic phosphate/pyrophosphate ratio or reducing inflammatory mediators [15,16]. Furthermore, inflammation itself suppresses osteoblast activity and ALP levels [19,20,21], and allopurinol’s anti-inflammatory properties [21] may indirectly contribute to a more favorable environment for bone formation and remodeling. Kanczler et al. [19] specifically showed that XO mediates cytokine-induced bone resorption, a process inhibited by allopurinol, further consistent with a potential direct role for XO inhibition in bone turnover beyond urate lowering. These findings provide a plausible context for the observed association between allopurinol treatment and increased alkaline phosphatase activity, potentially involving the bone isoform, within the scope of bone metabolism.

Although ALP increases are also linked to cardiovascular death, allopurinol was not significantly associated with the composite outcome of renal and patient death in this cohort (HR 1.32, 95% CI: 0.87–1.99); however, the wide confidence interval is consistent with both a clinically meaningful increase and a clinically meaningful decrease in risk, and a true association cannot be excluded. The composite endpoint of renal and patient death combines two clinically distinct events with potentially different predictors. Renal death reflects primarily CKD progression, whereas patient death encompasses cardiovascular, infectious, and other causes. The limited number of events precluded separate analyses of each component, which represents a limitation that future, larger studies should address. This may reflect the possibility that any detrimental effects associated with allopurinol-associated increases in ALP could theoretically be offset by potential benefits of reduced XO activity, though this remains speculative. The previously discussed effects of reduced oxidative stress and our recent demonstration of lowered low-density lipoprotein cholesterol in CKD patients treated with allopurinol are two potential associations of XO inhibition that warrant further study [22]. These complexities highlight the challenges of determining mortality risk with the use of allopurinol in this population.

While aspartate aminotransferase (AST) levels were higher in the allopurinol group at baseline, they remained within the normal physiological range for over 95% of patients in both groups. Moreover, AST was included as a covariate in all longitudinal ALP sub-models, and the allopurinol–ALP association persisted after this adjustment throughout follow-up, providing further evidence against a hepatic source. Similarly, the observed interactions between allopurinol with PTH and urate are not readily explained by hepatic injury as the predominant source of the observed ALP increase. Furthermore, our observation of a PTH increase with allopurinol use aligns with the findings of Kohri et al., who reported that long-term allopurinol treatment increased PTH without elevating hepatocellular enzymes [23]. The exclusion of patients with a history of liver disease from the analytic cohort further reduces the probability of hepatic isoform predominance. Additionally, FGF23, a hormone predominantly secreted by osteocytes, demonstrated an independent and consistent positive association with ALP across all models, in keeping with a shared bone mineral metabolism axis. These converging lines of evidence raise the hypothesis of a bone-predominant contribution to the observed ALP elevation. However, because bone-specific ALP isoforms, bone turnover markers (e.g., PINP, CTX), and bone histology were not evaluated, this interpretation remains speculative.

A notable finding from our study is the association between allopurinol use and PTH levels. PTH plays a complex, dual role in bone: intermittent exposure promotes osteoblast activity and bone formation, while chronic elevation favors bone resorption and leads to high-turnover bone disease in CKD [24,25,26]. The observed PTH increase with allopurinol, similar to reports by Kohri et al. [23], might represent a compensatory response. If the observed increase in total ALP is presumed to reflect, at least in part, enhanced osteoblast activity or altered bone turnover, this could theoretically lead to subtle shifts in calcium homeostasis [27]. However, the chronic implications of this PTH elevation in the context of CKD-MBD warrant further investigation.

Our analyses also revealed a significant negative interaction between allopurinol and PTH in the prediction of ALP. Both the interaction term in the GSEM and the controlled direct effect analysis consistently demonstrated that the positive association between allopurinol and ALP was most pronounced at lower PTH levels and progressively weakened as PTH increased. This suggests that in PTH-inferred high bone turnover states, the potent effects of PTH-driven bone resorption may overshadow or modify allopurinol’s association with ALP. While elevated ALP in hyperparathyroidism is often attributed to increased bone turnover (both formation and resorption), our findings suggest that any potential allopurinol-associated changes in bone metabolism might be more evident in conditions of lower baseline bone remodeling activity. The controlled direct effects analysis illustrates how the allopurinol and ALP association varied across PTH strata, showing a more pronounced relationship at lower PTH-inferred bone turnover. Previous work suggesting that oxidative stress reduction can mitigate PTH-driven bone resorption provides another potential layer of complexity in this interaction [12,13,14,15,16]. Whether this observed interaction reflects a protective adaptation against bone loss or a complex interplay in regulating bone turnover remains an important question.

Urate had a small and non-significant indirect effect on ALP, indicating that allopurinol’s association with ALP was not primarily attributable to its urate-lowering effect. PTH demonstrated a modest but statistically significant mediation, accounting for only 7.9% of allopurinol’s total effect on ALP. This raises the possibility that other factors—potentially the direct anti-oxidative or anti-inflammatory effects of allopurinol—may contribute to allopurinol’s association with ALP levels [12,13].

**Strengths and Limitations:** A major strength of this study was its access to a large, well-characterized multi-ethnic CKD cohort with many relevant longitudinal data including ALP, PTH, FGF23, and 25(OH)D. The mediation analyses assume no unmeasured confounding of the exposure–mediator or mediator–outcome relationships, the so-called sequential ignorability assumption, which cannot be verified in observational data. Therefore, all mediation findings presented in this study are strictly **exploratory and hypothesis-generating** and should not be misconstrued as definitive proof of biological pathways. Allopurinol prescriptions were written by the study nephrologists and exposure status was systematically recorded at every six-monthly visit, ensuring reliable capture of changes in allopurinol use, including initiation, continuation, and discontinuation, throughout follow-up. However, multi-ethnic CKD patients in Canada may differ from CKD patients in other regions, especially where healthcare access or treatment practices differ. Nonetheless, the sample is diverse enough to offer some insights that extend across various ethnic groups. Another strength was the use of generalized structural equation models which permitted simultaneous evaluation of longitudinal and survival outcomes. Finally, the use of causal mediation and controlled direct effects analyses allowed us to explore potential mechanistic pathways.

Several limitations require mention. As a post hoc analysis of the CAN-AIM to PREVENT, our study results are hypothesis-generating and do not permit causal inference. The allopurinol dose was not recorded in the parent study, precluding examination of dose–response relationships. There were no bone biopsy or fracture, and no vascular calcification data to support definitive conclusions about the specific impact of allopurinol on bone formation and fracture risk, and vascular mineralization respectively. The limited number of hard events and relatively short follow-up may also hinder the evaluation of relationships between allopurinol and bone, vascular, renal and mortality outcomes. The absence of bone-specific ALP isoform measurements (to permit differentiation from other isoforms of ALP) and other direct markers of bone formation (e.g., procollagen type I N-terminal propeptide [PINP], osteocalcin) or resorption (e.g., C-terminal telopeptide of type I collagen [CTX]) represents another limitation. Finally residual confounding by unmeasured factors (e.g., dietary phosphate intake, genetic variations, physician prescribing patterns, allopurinol adherence) remained a possibility that could not be ruled out.

**Clinical Implications:** Given its critical role in promoting bone and vascular mineralization, we believe our observation of increased total ALP levels associated with allopurinol use deserves further study in this and other populations. Future research could consider randomized controlled trials to prospectively assess the associations between allopurinol and bone mineral metabolism, fracture risk, and vascular outcomes, and incorporate bone histomorphometry and vascular calcification scores to clarify whether any bone-isoform component of the observed ALP elevation is associated with bone turnover outcomes and/or vascular calcification. Mechanistic studies could explore whether and how XO inhibition is associated with PTH and ALP changes, including potential direct effects on parathyroid glands or bone cells. Finally, studies examining dose-dependent effects of allopurinol and the impact of achieving specific urate or ALP targets on bone outcomes could also be considered.

**Conclusions:** The use of allopurinol was prospectively associated with increased ALP but not renal or patient death in patients with non-dialysis CKD. Allopurinol’s association with ALP was the strongest at lower urate and PTH levels. Future studies could evaluate the mechanisms of these findings and determine their clinical significance and generalizability to other populations.

## Figures and Tables

**Table 1:** Participant characteristics at study baseline.

Variable *	Total (*n* = 1636)	Not on Allopurinol (*n* = 1248)	On Allopurinol (*n* = 388)	*p*-Value
Age (Years) ^a^	70.0 ± 11.6	69.7 ± 11.9	70.9 ± 10.9	0.1778
Male (%) ^b^	66.2	62.5	77.3	0.000
White ethnicity (%) ^b^	57.9	50.0	63.7	0.006
Vitamin D supplements (%) ^b^	22.3	21.0	26.3	0.029
History of diabetes (%) ^b^	49.0	50.6	44.1	0.026
History of hypertension (%) ^b^	93.7	93.6	94.1	0.733
History of coronary artery disease (%) ^b^	25.7	25.4	26.6	0.652
Other history of heart disease (%) ^b^	23.4	23.5	23.2	0.909
History of peripheral artery disease (%) ^b^	11.9	12.3	10.8	0.446
History of stroke (%) ^b^	8.9	9.0	8.5	0.776
eGFR (ml/min/1.73 m^2^) ^a^	38.8 ± 11.2	39.2 ± 11.3	37.6 ± 10.8	0.0156
CRP (mg/L) ^c^	3.7 ± 7.3	3.6 ± 7.6	3.9 ± 6.2	0.0123
Urate (µmol/L) ^a^	446.8 ± 112.3	464.2 ± 110.4	391.0 ± 99.8	<0.001
AST (U/L) ^c^	23.9 ± 10.9	23.3 ± 8.7	25.7 ± 15.6	0.0003
ALP (IU/L) ^c^	77.9 ± 34.7	76.6 ± 34.7	82.1 ± 34.4	0.0001
FGF23(RU/mL) ^c^	175.2 ± 361.9	181.6 ± 408.0	154.8 ± 128.1	0.8710
25(OH)D (nmol/L) ^c^	67.5 ± 30.8	67.4 ± 31.5	67.7 ± 28.3	0.6471
PTH (pmol/L) ^c^	6.4 ± 4.5	6.5 ± 4.6	6.4 ± 4.1	0.6980
Phosphate (mmol/L) ^a^	1.17 ± 0.22	1.17 ± 0.21	1.18 ± 0.23	0.6192
Calcium (mmol/L) ^a^	2.31 ± 0.11	2.30 ± 0.11	2.31 ± 0.11	0.0040

* Mean ± SD or frequency. **Abbreviations:** eGFR: Estimated Glomerular Filtration Rate; CRP: C-reactive protein; AST: Aspartate Aminotransferase; ALP: Alkaline Phosphatase; FGF23: Fibroblast Growth Factor23; PTH: Parathyroid Hormone. Statistical comparisons were performed using the following tests: ^a^ independent samples *t*-test (Age, eGFR, Urate, Phosphate, Calcium); ^b^ chi-square test (male sex, white ethnicity, Vitamin D supplementation, history of diabetes, hypertension, coronary artery disease, other heart disease, peripheral artery disease, and stroke), ^c^ Wilcoxon rank-sum test (CRP, AST, ALP, FGF23, PTH, 25(OH)D). Statistical significance was defined as a two-sided *p*-value < 0.05.

**Table 2:** Allopurinol use as a predictor of alkaline phosphatase and censored events (renal and patient deaths) using GSEM.

	Model 1		Model 2	
Outcome/Variable	% Change or HR (95% CI)	*p*-Value	% Change or HR (95% CI)	*p*-Value
Change in Alkaline Phosphatase (% *)				
Allopurinol use (yes)	18.29 (10.90–26.19)	<0.001	11.83 (5.55–18.48)	<0.001
Natural log PTH	18.13 (16.50–19.80)	<0.001	–	–
PTH tertile 2 vs. 1	–	–	9.73 (7.85–11.66)	<0.001
PTH tertile 3 vs. 1	–	–	22.81 (20.30–25.36)	<0.001
Allopurinol × PTH interaction	−3.83 (−6.09–−1.52)	0.001	–	–
Allopurinol × PTH tertile 2 vs. 1 interaction	–	–	0.22 (−3.04–3.60)	0.895
Allopurinol × PTH tertile 3 vs. 1 interaction	–	–	−3.83 (−7.17–−0.37)	0.031
Urate	−0.005 (−0.012–0.002)	0.183	−0.00 (−0.01–0.00)	0.222
Allopurinol × Urate interaction	−0.013 (−0.026–0.000)	0.044	−0.017 (−0.03–0.00)	0.037
FGF23	2.10 (1.01–3.20)	<0.001	2.71 (1.57–3.86)	<0.001
25(OH)D	1.37 (−0.33–3.11)	0.114	−0.07 (−1.75–1.63)	0.932
Renal and patient deaths (HR **)				
Allopurinol use (yes)	1.32 (0.87–1.99)	0.193	1.29 (0.85–1.96)	0.238
Natural log ALP	2.09 (1.23–3.57)	0.007	2.01 (1.17–3.44)	0.012
Urate	1.00 (1.00–1.00)	0.988	1.00 (0.99–1.00)	0.936
Phosphate	0.98 (0.50–1.93)	0.949	1.02 (0.52–2.03)	0.946
Natural log PTH	1.07 (0.75–1.53)	0.690	–	–
PTH tertile 2 vs. 1	–	–	1.11 (0.65–1.89)	0.711
PTH tertile 3 vs. 1	–	–	1.40 (0.78–2.50)	0.258
Natural log FGF23	2.24 (1.81–2.78)	<0.001	2.23 (1.80–2.77)	<0.001
Natural log 25(OH)D	0.93 (0.60–1.45)	0.748	0.97 (0.63–1.50)	0.893

**Adjustments for change in alkaline phosphatase:** season, aspartate aminotransferase, C-reactive protein (CRP), body mass index, sex, ethnicity, estimated glomerular filtration rate (eGFR), age, diabetes status, visit number (used as a proxy for time), vitamin D usage, and the quadratic term of visit number. **Adjustments for renal and patient survival:** CRP, presence of other heart disease, peripheral arterial disease, cerebrovascular disease, coronary artery disease, estimated glomerular filtration rate (eGFR), sex, ethnicity, phosphate, hypertension, diabetes mellitus, and body mass index. * Percentage change in alkaline phosphatase calculated as, where (e^β^ − 1) × 100) represents the model-predicted beta coefficient from the longitudinal sub-model of the joint GSEM. ** Hazard ratios (HR) derived from the time-to-event sub-model of the joint GSEM, modeled using a Weibull family with a log link function and adjusted for right-censoring, representing exponentiated beta coefficients (e^β^). **Abbreviations:** ALP: Alkaline Phosphatase, FGF23: Fibroblast Growth Factor-23, HR: Hazard Ratio, PTH: Parathyroid Hormone, 25(OH)D: 25-Hydroxyvitamin D, log: natural logarithm.

**Table 3:** Allopurinol use, urate, and bone mineral metabolism markers as predictors of bone mineral marker levels using GSEM.

	Model 3		Model 4	
Independent Variable	PTH % Difference (95% CI)	*p*-Value	FGF23 % Difference (95% CI)	*p*-Value
Allopurinol (Yes)	27.05 (15.92–39.23)	<0.001	3.32 (−7.23–15.04)	0.552
Urate	0.06 (0.04–0.07)	<0.001	0.03 (0.01–0.04)	0.001
Allopurinol × Urate Interaction	−0.05 (−0.07–−0.03)	<0.001	−0.01 (−0.04–0.01)	0.413
Allopurinol × PTH Interaction	–	–	–	–
Natural log FGF23	13.69 (11.58–15.84)	<0.001	–	–
Natural log PTH	–	–	20.99 (17.55–24.51)	<0.001
Natural log 25(OH)D	−29.03 (−30.83–−27.19)	<0.001	5.63 (2.16–9.24)	0.001
Calcium	−74.91 (−78.28–−71.00)	<0.001	122.86 (94.10–155.70)	<0.001
Phosphate	−14.41 (−19.14–−9.42)	<0.001	83.04 (69.16–98.08)	<0.001
Renal and patient deaths (HR **)				
Natural log ALP	2.09 (1.23–3.57)	0.007	2.09 (1.23–3.57)	0.007
Allopurinol (Yes)	1.32 (0.87–1.99)	0.193	1.32 (0.87–1.99)	0.193
Urate	1.0 (1.0–1.0)	0.988	1.00 (0.998–1.002)	0.988
Natural log PTH	1.07 (0.75–1.53)	0.690	1.07 (0.75–1.53)	0.690
Natural log FGF23	2.24 (1.81–2.78)	<0.001	2.24 (1.81–2.78)	<0.001
Natural log 25(OH)D	0.93 (0.60–1.45)	0.748	0.93 (0.60–1.45)	0.748
Phosphate	0.98 (0.50–1.93)	0.949	0.98 (0.50–1.93)	0.949

**Note:** Bone mineral marker levels adjusted for season, calcium and phosphate as appropriate, aspartate aminotransferase, C-reactive protein (CRP), body mass index (BMI), sex, ethnicity, eGFR, age, diabetes status, visit number (used as a proxy for time), vitamin D usage, and the quadratic term of visit number. Differences in variable levels expressed as percentages and calculated using exponentials of model-predicted beta coefficients: (e^β^ − 1) × 100). Differences in censored events expressed as hazard ratios and calculated by exponentiating beta coefficients (e^β^). ** Hazard ratios (HR) derived from the time-to-event sub-model of the joint GSEM, modeled using a Weibull family with a log link function and adjusted for right-censoring, representing exponentiated beta coefficients (e^β^). **Abbreviations:** ALP: Alkaline Phosphatase, FGF23: Fibroblast Growth Factor-23, HR: Hazard Ratio, PTH: Parathyroid Hormone, 25(OH)D: 25-Hydroxyvitamin D, log: Logarithm.

**Table 4:** Mediation analysis.

A Treatment	B Mediator	C Outcome	Indirect Effect (95% CI) *p*-Value	Direct Effect (95% CI) *p*-Value	Total Effect	Mediation Proportion (%) (95% CI) *p*-Value
Allopurinol	Urate	ALP	0.008 (−0.00, 0.02) 0.077	0.05 (0.04, 0.07) <0.001	0.06 (0.05, 0.08) <0.001	12.9 (−0.02, 0.27) 0.085
Allopurinol	PTH	ALP	0.005 (0.001, 0.009) 0.007	0.06 (0.04, 0.07) <0.001	0.06 (0.05, 0.08) <0.001	7.9 (2.3, 13.7) 0.006
Allopurinol	Urate	PTH	−0.03 (−0.05, −0.01) 0.001	0.07 (0.03, 0.10) <0.001	0.04 (0.01, 0.07) 0.006	N/A
Allopurinol	Urate	25(OH)D	0.05 (0.04, 0.06), <0.001	−0.052 (−0.073, −0.031), <0.001	−0.006 (−0.02, 0.01), 0.500	N/A

**Abbreviations:** ALP: Alkaline Phosphatase, PTH: Parathyroid Hormone, 25(OH)D: 25-Hydroxyvitamin D, N/A: Not applicable. When direct and indirect effects are in opposing directions (inconsistent mediation), the proportion mediated metric loses its intuitive interpretability and is not recommended as a summary measure [18].

**Table 5:** Mediation analysis using controlled direct effects.

Treatment	Outcome	Mediator	Mediator Level	Proportion (95% CI), *p*-Value
Allopurinol	ALP	PTH	2.69 (pmol/L)	8.5 (6.2, 10.7), <0.001
			3.60 (pmol/L)	7.38 (5.61, 9.15), <0.001
			5.30 (pmol/L)	5.89 (4.46, 7.32), <0.001
			7.9 (pmol/L)	4.4 (2.7, 6.0), <0.001
			11.9 (pmol/L)	2.8 (0.4, 5.1), 0.022
Allopurinol	ALP	Urate	298 (μmol/L)	10.90 (8.7, 13.1), <0.001
			361 (μmol/L)	8.74 (7.07, 10.42), <0.001
			434 (μmol/L)	6.24 (4.61, 7.86), <0.001
			507 (μmol/L)	3.74 (1.59, 5.88), 0.001
Allopurinol	PTH	Urate	Very Low (298 μmol/L)	24.1 (20.2, 27.9), <0.001
			Low (361 μmol/L)	17.16 (14.19, 20.12), <0.001
			Median (434 μmol/L)	9.16 (6.16, 12.17), <0.001
			High (507 μmol/L)	1.1 (−2.9, 5.2), 0.579

Cutoffs correspond to the 10th, 25th, 50th, 75th, and 90th percentiles of the PTH distribution, and the 10th, 25th, 50th, and 75th percentiles of the urate distribution in the analytic cohort. For this table, an antilog transformation was performed to obtain the actual PTH values using the formula: PTH = e^ln(PTH)^. **Abbreviations:** ALP: Alkaline Phosphatase, PTH: Parathyroid Hormone.

## Data Availability

The data that support the findings of this study are available from the corresponding author—[T.S.], upon reasonable request.

## Supplementary Materials

The following supporting information can be downloaded at https://www.mdpi.com/article/10.3390/jcm15124685/s1, Figure S1: Analytic Workflow; Table S1: Sensitivity Analysis—Allopurinol use as a predictor of Alkaline Phosphatase and censored events (renal and patient deaths) using GSEM, restricted to participants with AST ≤ 40 U/L (9799 observation).

## Abbreviations

The following abbreviations are used in this manuscript:

25(OH)D	25-Hydroxyvitamin D
ALP	Alkaline Phosphatase
AST	Aspartate Aminotransferase
BALP	Bone-specific Alkaline Phosphatase
BMI	Body Mass Index
CI	Confidence Interval
CKD	Chronic Kidney Disease
CKD-MBD	Chronic Kidney Disease–Mineral and Bone Disorder
CRP	C-Reactive Protein
CTX	C-Terminal Telopeptide of Type I Collagen
eGFR	Estimated Glomerular Filtration Rate
FGF23	Fibroblast Growth Factor-23
GSEM	Generalized Structural Equation Modeling
HR	Hazard Ratio
IL-1β	Interleukin-1 Beta
KDIGO	Kidney Disease: Improving Global Outcomes
LDL	Low-Density Lipoprotein
PINP	Procollagen Type I N-Terminal Propeptide
PTH	Parathyroid Hormone
ROS	Reactive Oxygen Species
SD	Standard Deviation
TNAP	Tissue Non-specific Alkaline Phosphatase
TNFα	Tumor Necrosis Factor Alpha
XO	Xanthine Oxidase
