# Supplementary material for: Allopurinol and Alkaline Phosphatase Levels in Patients with Non-Dialysis CKD
Source: J Clin Med. 2026 Jun 17;15(12):4685. doi: 10.3390/jcm15124685 (PMC13302618; doi:10.3390/jcm15124685)
Supplement: Supplementary file 1 [file jcm-15-04685-s001.zip › jcm-4338601-supplementary.pdf]

Supplementary Material

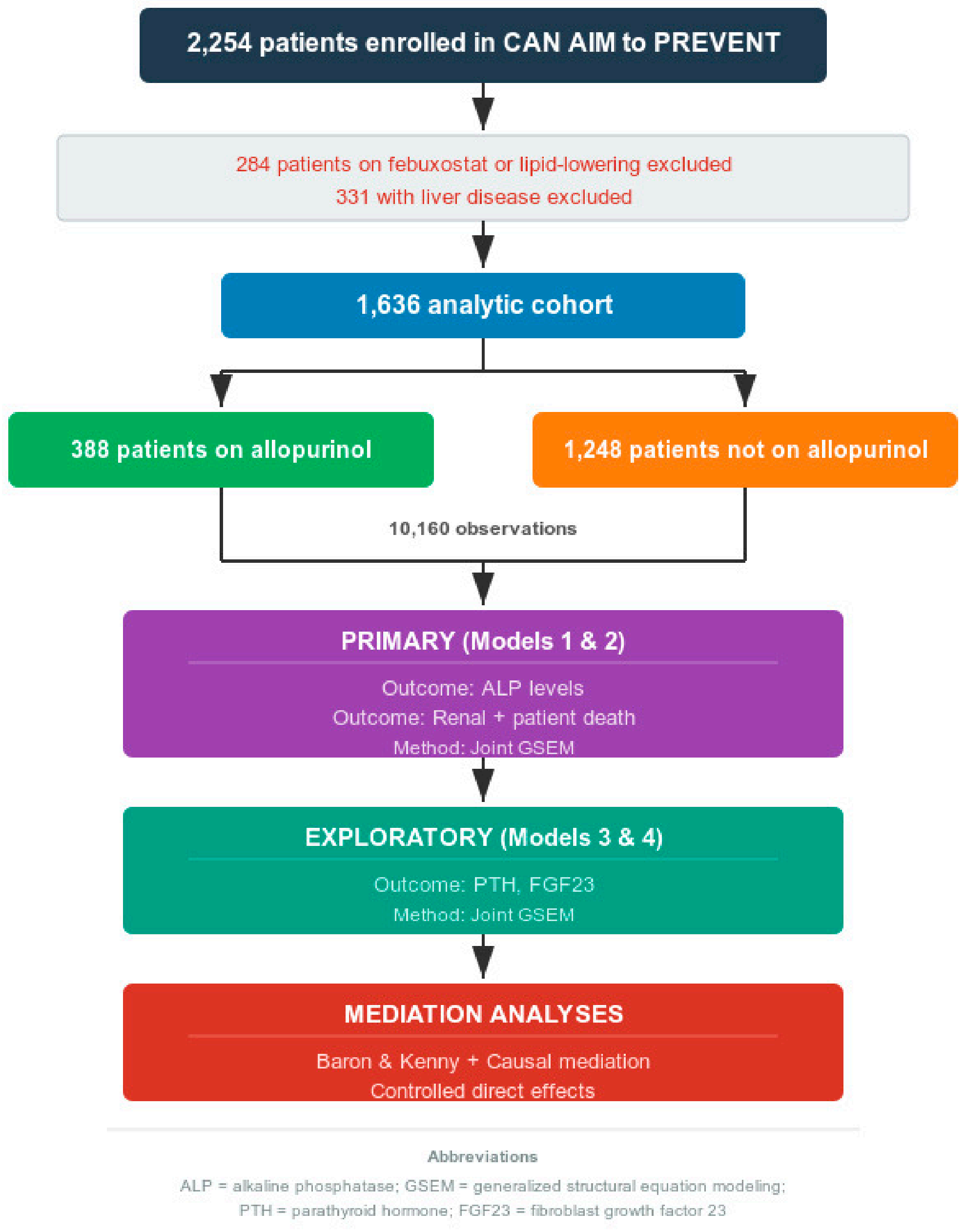

Figure S1. Analytic Workflow

Table S1: Sensitivity Analysis - Allopurinol use as a predictor of Alkaline Phosphatase and censored events (renal and patient deaths) using GSEM, restricted to participants with AST ≤ 40 U/L (9,799 observation)

**Outcome / Variable**	**Model 1**<br>**% change or HR (95% CI)**	**p value**	**Model 2**<br>**% change or HR (95% CI)**	**p value**
**Change in Alkaline Phosphatase (%*)**				
Allopurinol use (yes)	19.09 (11.63 – 27.07)	< 0.001	13.24 (6.86 – 20.00)	< 0.001
Natural log PTH	17.76 (16.12 – 19.40)	< 0.001	–	–
PTH tertile 2 vs 1	–	–	9.49 (7.64 – 11.37)	< 0.001
PTH tertile 3 vs 1	–	–	22.34 (19.88 – 24.82)	< 0.001
Allopurinol × PTH interaction	−3.68 (−5.96 – −1.35)	0.002	–	–
Allopurinol × PTH tertile 2 vs 1 interaction	–	–	−0.78 (−3.99 – 2.54)	0.640
Allopurinol × PTH tertile 3 vs 1 interaction	–	–	−3.82 (−7.18 – −0.33)	0.032
Urate	−0.004 (−0.011 – 0.004)	0.337	−0.003 (−0.010 – 0.004)	0.398
Allopurinol × Urate interaction	−0.016 (−0.029 – −0.003)	0.019	−0.016 (−0.029 – −0.003)	0.017
FGF23	2.27 (1.16 – 3.39)	< 0.001	2.96 (1.84 – 4.09)	< 0.001
25(OH)D	1.18 (−0.54 – 2.92)	0.180	−0.12 (−1.81 – 1.60)	0.890
**Renal and patient deaths (HR**)**				
Allopurinol use (yes)	1.41 (0.91 – 2.17)	0.120	1.36 (0.88 – 2.12)	0.170
Natural log ALP	1.96 (1.08 – 3.54)	0.027	1.88 (1.03 – 3.41)	0.039
Urate	1.00 (1.00 – 1.00)	0.923	1.00 (1.00 – 1.00)	0.971
Phosphate	0.98 (0.48 – 2.01)	0.961	1.05 (0.51 – 2.15)	0.898
Natural log PTH	1.19 (0.82 – 1.74)	0.366	–	–

PTH tertile 2 vs 1	–	–	1.02 (0.58 – 1.81)	0.940
PTH tertile 3 vs 1	–	–	1.56 (0.85 – 2.86)	0.149
Natural log FGF23	2.19 (1.75 – 2.74)	< 0.001	2.19 (1.76 – 2.73)	< 0.001
Natural log 25(OH)D	0.99 (0.63 – 1.57)	0.974	1.02 (0.65 – 1.61)	0.918

Adjustments for change in Alkaline Phosphatase: season, aspartate aminotransferase, C-reactive protein (CRP), body mass index, sex, ethnicity, estimated glomerular filtration rate (eGFR), age, diabetes status, visit number (used as a proxy for time), vitamin D usage, and the quadratic term of visit number.

Adjustments for renal and patient survival: CRP, presence of other heart disease, peripheral arterial disease, cerebrovascular disease, coronary artery disease, estimated glomerular filtration rate (eGFR), sex, ethnicity, phosphate, hypertension, diabetes mellitus, and body mass index.

*Percentage change in Alkaline Phosphatase calculated as, where (e^{β} - 1) * 100) represents the model-predicted beta-coefficient from the longitudinal sub-model of the joint GSEM.

** Hazard Ratios (HR) derived from the time-to-event sub-model of the joint GSEM, modeled using a Weibull family with a log link function and adjusted for right-censoring, representing exponentiated beta-coefficients (e^{β}).

Abbreviations: ALP: Alkaline Phosphatase, FGF23: Fibroblast Growth Factor-23, HR: Hazard Ratio, PTH: Parathyroid Hormone, 25(OH)D: 25-Hydroxyvitamin D, log: natural logarithm.
